# Supplementary material for: Meiotic Cas9 expression mediates gene conversion in the male and female mouse germline
Source: PLoS Biol. 2021 Dec 23;19(12):e3001478. doi: 10.1371/journal.pbio.3001478 (PMC8699911; doi:10.1371/journal.pbio.3001478)
Supplement: S1 Fig — (A) SnapGene schematic of P2A-Cas9-T2A-eGFP integrated into the 3′ end of the endogenous Spo11 locus. LR, Left Homology Arm; RR, Right Homology Arm. Maroon arrows (1 to 7) represent aligned Sanger sequencing traces that tile the knock-in. Dark blue arrows (LHom/RHom) represent aligned Sanger sequencing traces that span the homology arm and part of the knock-in construct. Vertical dotted lines show that the primer binding sites are outside of the homology arm and inside of the synthetic construct. (B) PCR amplification of the left and right homology arm. All primer sequences, PCR conditions, and amplicon lengths are in S1 Table. The validated knock-in allele sequence is available at GenBank under accession number OK067273. Sanger sequencing files can be found at the associated Zenodo data repository (https://doi.org/10.5281/zenodo.5510697) in folder “Spo11_Cas9_knockin_validation.” The raw gel image in (B) can also be found at the Zenodo data repository labeled “S1 Raw Images.” (PDF) [file pbio.3001478.s001.pdf]

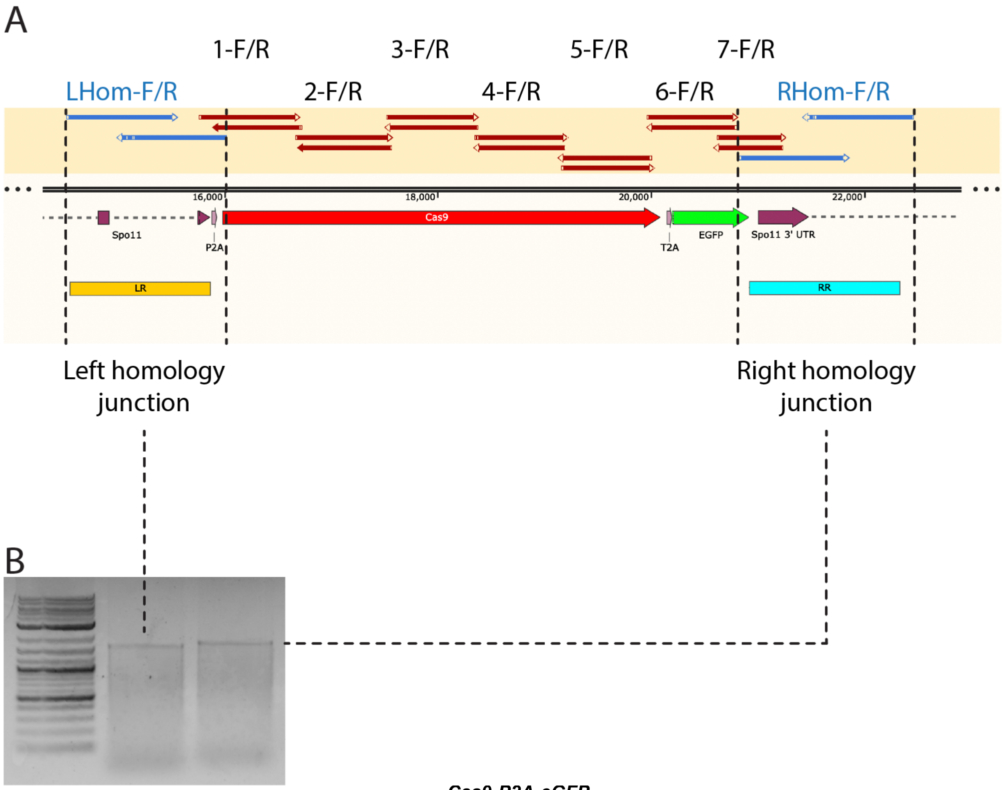

S1 Fig. PCR validation of *Spo11*$^{Cas9\text{-}P2A\text{-}eGFP}$ integration.

(A) SnapGene schematic of P2A-Cas9-T2A-eGFP integrated into the 3' end of the endogenous *Spo11* locus. LR, Left Homology Arm; RR, Right Homology Arm. Maroon arrows (1-7) represent aligned Sanger sequencing traces that tile the knock-in. Dark blue arrows (LHom/RHom) represent aligned Sanger sequencing traces that span the homology arm and part of the knock-in construct. Vertical dotted lines show that the primer binding sites are outside of the homology arm and inside of the synthetic construct. **(B)** PCR amplification of the left and right homology arm. All primer sequences, PCR conditions, and amplicon lengths are in S1 Table. The validated knock-in allele sequence is available at GenBank under accession number OK067273. Sanger sequencing files can be found at the associated Zenodo data repository (https://doi.org/10.5281/zenodo.5510697) in folder 'Spo11_Cas9_knockin_validation'. The raw gel image in (B) can also be found at the Zenodo data repository labeled 'S1_Raw_Images.pdf'.
