# Supplementary material for: Meiotic Cas9 expression mediates gene conversion in the male and female mouse germline
Source: PLoS Biol. 2021 Dec 23;19(12):e3001478. doi: 10.1371/journal.pbio.3001478 (PMC8699911; doi:10.1371/journal.pbio.3001478)
Supplement: S2 Fig — (A–C) Western blot of (A) SPO11, (B) CAS9, and (C) eGFP on 3 wild-type and 3 Spo11Cas9-P2A-eGFP/+ adult testes. (A’–C’) Serially-stained membranes adding anti β-ACTIN to detect the loading control. PP, Precision Plus Ladder; MM, MagicMark Ladder. The raw gel images can be found at the associated Zenodo data repository (https://doi.org/10.5281/zenodo.5510697) in the file labeled “S1 Raw Images.pdf.” (PDF) [file pbio.3001478.s002.pdf]

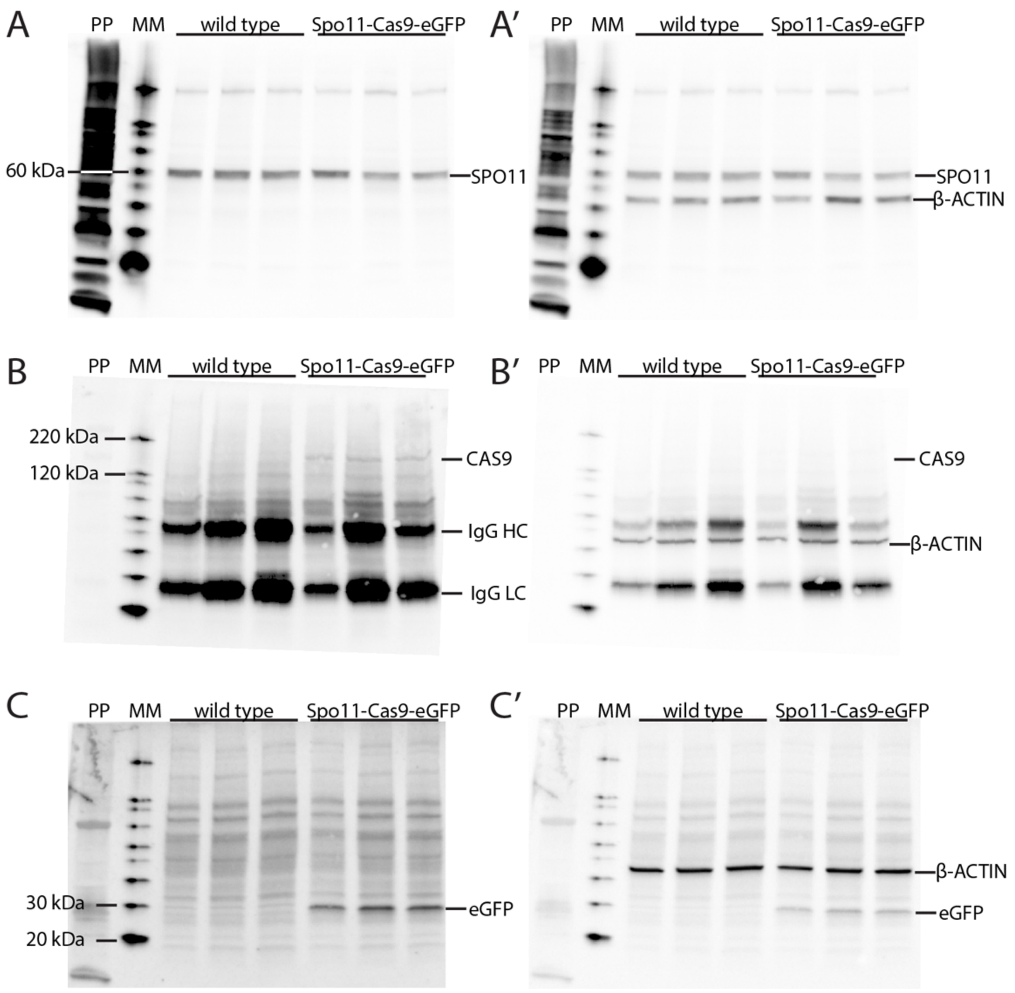

S2 Fig. Western blot of SPO11, CAS9, and eGFP in adult testis.
(A-C) Western blot of (A) SPO11, (B) CAS9, and (C) eGFP on three wild type and three *Spo11*$^{Cas9\text{-}P2A\text{-}eGFP/+}$ adult testes. **(A'-C')** Serially-stained membranes adding anti β-ACTIN to detect the loading control. PP, Precision Plus Ladder; MM, MagicMark Ladder. The raw gel images can be found at the associated Zenodo data repository (https://doi.org/10.5281/zenodo.5510697) in the file labeled 'S1_Raw_Images.pdf'.
