# Supplementary material for: Meiotic Cas9 expression mediates gene conversion in the male and female mouse germline
Source: PLoS Biol. 2021 Dec 23;19(12):e3001478. doi: 10.1371/journal.pbio.3001478 (PMC8699911; doi:10.1371/journal.pbio.3001478)
Supplement: S3 Fig — (A) Spo11Cas9-P2A-eGFP/+ and (B) Spo11Cas9-P2A-eGFP/Cas9-P2A-eGFP seminiferous tubules; scale bar is 50 μm. Cells in seminiferous tubules in (B) likely do not complete meiosis, evidenced by absence of sperm. (C) Tiled image of Spo11Cas9-P2A-eGFP/Cas9-P2A-eGFP testis; scale bar is 200 μm. All seminiferous tubules are deformed and devoid of sperm. (PDF) [file pbio.3001478.s003.pdf]

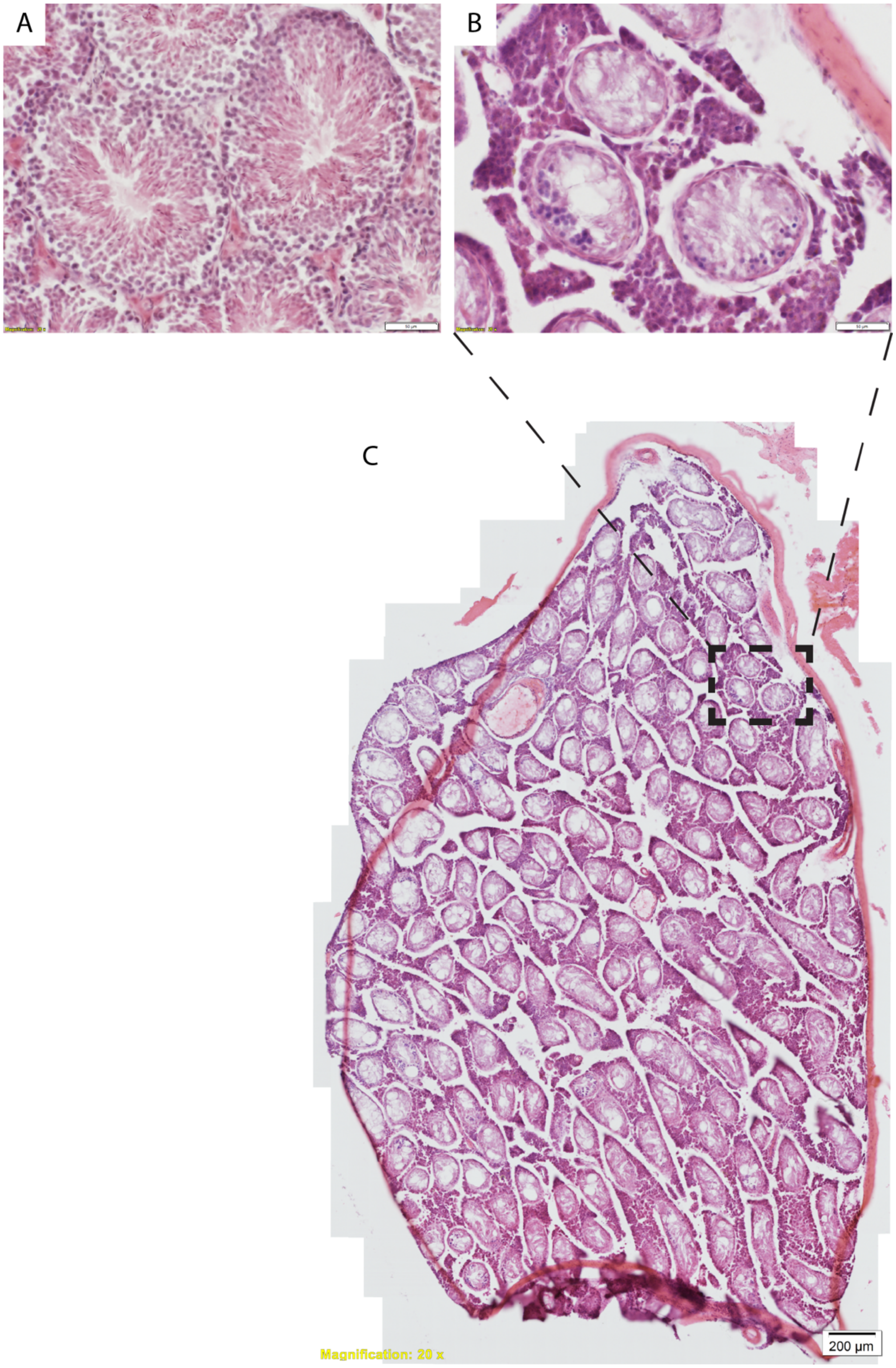

S3 Fig. Hematoxylin and eosin staining of testes.
(A) *Spo11*$^{Cas9\text{-}P2A\text{-}eGFP/+}$ and **(B)** *Spo11*$^{Cas9\text{-}P2A\text{-}eGFP/Cas9\text{-}P2A\text{-}eGFP}$ seminiferous tubules; scale bar is 50 µm. Cells in seminiferous tubules in (B) likely do not complete meiosis, evidenced by absence of sperm. **(C**) Tiled image of *Spo11*$^{Cas9\text{-}P2A\text{-}eGFP/Cas9\text{-}P2A\text{-}eGFP}$ testis; scale bar is 200 µm. All seminiferous tubules are deformed and devoid of sperm.
