# Supplementary material for: Meiotic Cas9 expression mediates gene conversion in the male and female mouse germline
Source: PLoS Biol. 2021 Dec 23;19(12):e3001478. doi: 10.1371/journal.pbio.3001478 (PMC8699911; doi:10.1371/journal.pbio.3001478)
Supplement: S4 Fig — (A-C) Wild-type embryonic ovary. All other panels in each row are Spo11Cas9-P2A-eGFP/+ littermates (e.g., A’–A”‘). (D) Wild-type and (D’) Spo11Cas9-P2A-eGFP/+ adult testis. Scale bar is 50 μm for all panels. Confocal fluorescence imaging settings are identical for all samples; eGFP expression is substantially higher in testes compared to any embryonic ovary time point. IF, immunofluorescence. (PDF) [file pbio.3001478.s004.pdf]

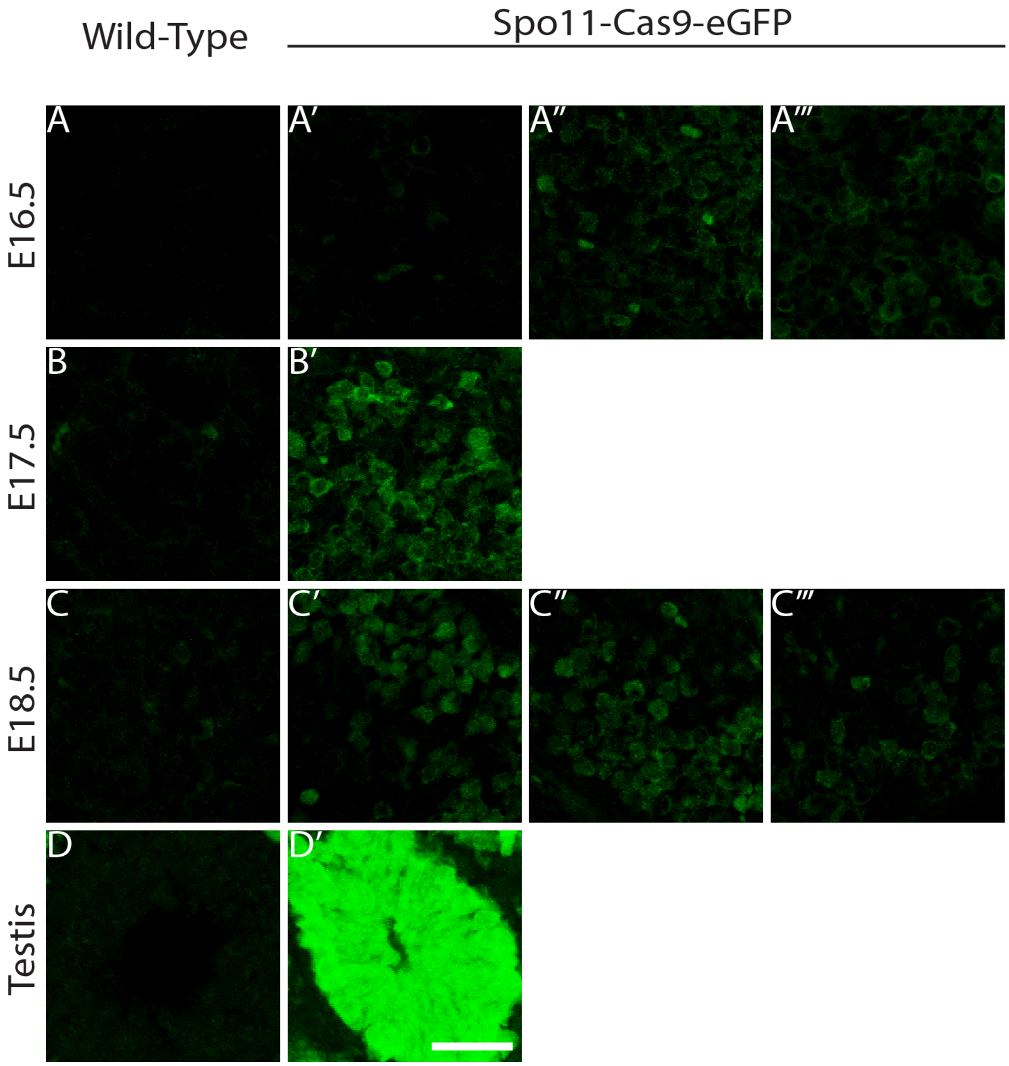

S4 Fig. Immunofluorescence detection of eGFP in embryonic ovaries and mature testis.
(A-C) Wild type embryonic ovary. All other panels in each row are *Spo11*$^{Cas9\text{-}P2A\text{-}eGFP/+}$ littermates (e.g. A'-A'''). **(D)** Wild type and **(D')** *Spo11*$^{Cas9\text{-}P2A\text{-}eGFP/+}$ adult testis. Scale bar is 50 μm for all panels. Confocal fluorescence imaging settings are identical for all samples; eGFP expression is substantially higher in testes compared to any embryonic ovary time point.
