# Supplementary material for: Meiotic Cas9 expression mediates gene conversion in the male and female mouse germline
Source: PLoS Biol. 2021 Dec 23;19(12):e3001478. doi: 10.1371/journal.pbio.3001478 (PMC8699911; doi:10.1371/journal.pbio.3001478)
Supplement: S5 Fig — (A) Tyrnull/Tyrnull tail tip. (B, C) TyrCopyCat/Tyrnull.tail tips. While clearly present or absent, mCherry expression varies in intensity between individuals of equivalent age. (PDF) [file pbio.3001478.s005.pdf]

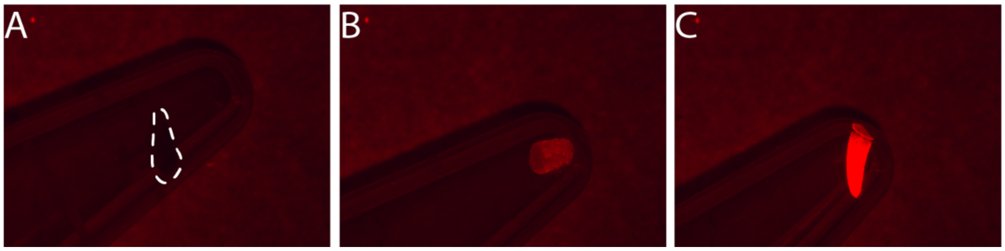

S5 Fig. Detection of CopyCat transgene by red fluorescent mCherry in tail tips.
(A) *Tyr*^{null}/*Tyr*^{null} tail tip. **(B-C)** *Tyr*^{CopyCat}/*Tyr*^{null}.tail tips. While clearly present or absent, mCherry expression varies in intensity between individuals of equivalent age.
