# Supplementary material for: Meiotic Cas9 expression mediates gene conversion in the male and female mouse germline
Source: PLoS Biol. 2021 Dec 23;19(12):e3001478. doi: 10.1371/journal.pbio.3001478 (PMC8699911; doi:10.1371/journal.pbio.3001478)
Supplement: S6 Fig — (A) Schematic of primer binding locations to detect presence of the TyrCopyCat transgene. (B) PCR confirmed presence of the TyrCopyCat transgene in each of the individuals marked as positive for a gene conversion event, with families of offspring from males and females numbered as in the table in Fig 2D. Two gels were merged for ease of understanding. The raw gel images in (B) can also at the associated Zenodo data repository (https://doi.org/10.5281/zenodo.5510697) in the file labeled “S1 Raw Images.” (PDF) [file pbio.3001478.s006.pdf]

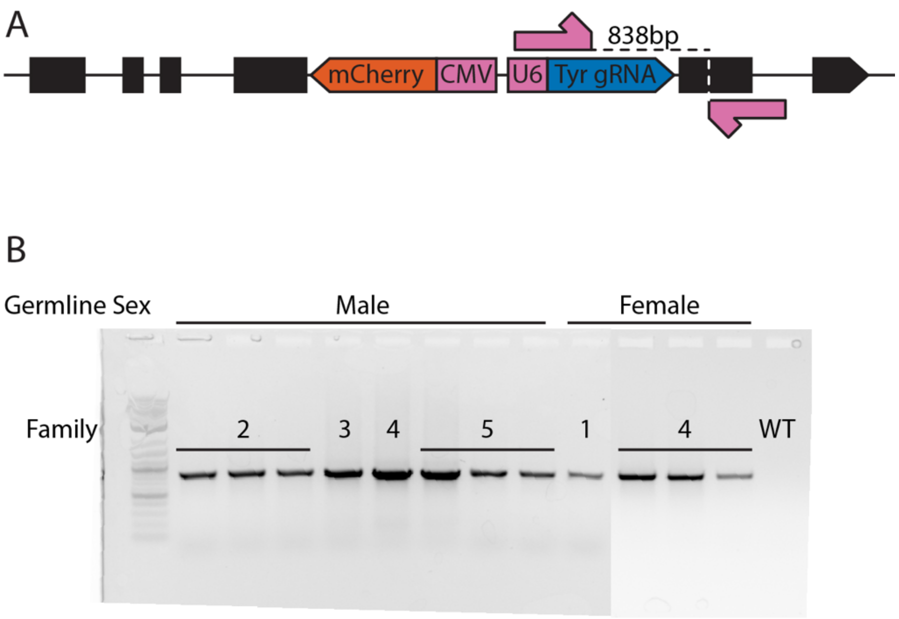

S6 Fig. PCR confirmation of *Tyr*^{CopyCat} in each offspring of an HDR event.
(A) Schematic of primer binding locations to detect presence of the *Tyr*^{CopyCat} transgene. **(B)** PCR confirmed presence of the *Tyr*^{CopyCat} transgene in each of the individuals marked as positive for a gene conversion event, with families of offspring from males and females numbered as in the table in Fig 2D. Two gels were merged for ease of understanding. The raw gel images in (B) can also at the associated Zenodo data repository (https://doi.org/10.5281/zenodo.5510697) in the file labeled ‘S1_Raw_Images.pdf’.
