# Supplementary material for: Meiotic Cas9 expression mediates gene conversion in the male and female mouse germline
Source: PLoS Biol. 2021 Dec 23;19(12):e3001478. doi: 10.1371/journal.pbio.3001478 (PMC8699911; doi:10.1371/journal.pbio.3001478)
Supplement: S7 Fig — (A) Schematic of primer binding location for PCR genotyping. (B) Gel depicting PCR of Spo11 locus revealing the genotype of Spo11+/+, Spo11Cas9-P2A-eGFP/+, and Spo11Cas9-P2A-eGFP/Cas9-P2A-eGFP. The raw gel image in (B) can be found at the associated Zenodo data repository (https://doi.org/10.5281/zenodo.5510697) in the file labeled “S1 Raw Images.” (PDF) [file pbio.3001478.s007.pdf]

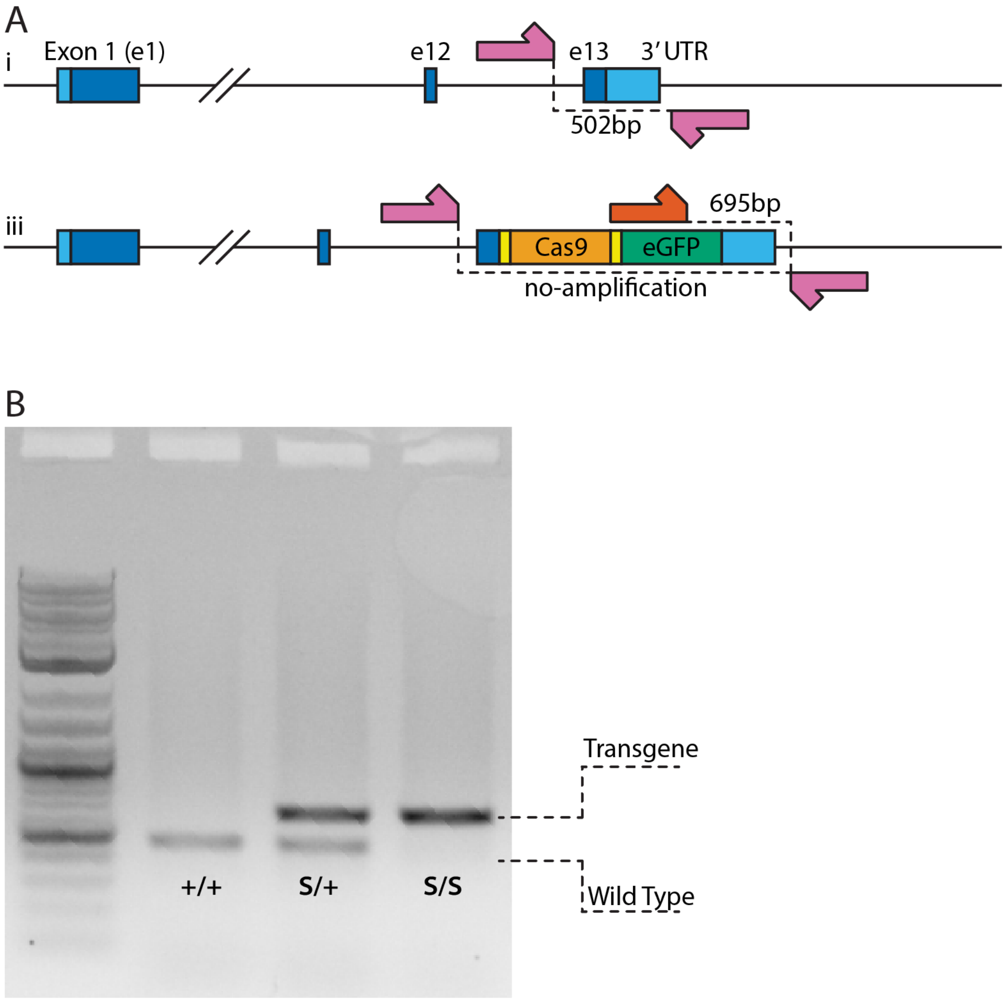

S7 Fig. Genotyping strategy for *Spo11*$^{Cas9\text{-}P2A\text{-}eGFP}$.
(A) Schematic of primer binding location for PCR genotyping. **(B)** Gel depicting PCR of Spo11 locus revealing the genotype of *Spo11*$^{+/+}$, *Spo11*$^{Cas9\text{-}P2A\text{-}eGFP/+}$, and *Spo11*$^{Cas9\text{-}P2A\text{-}eGFP/Cas9\text{-}P2A\text{-}eGFP}$. The raw gel image in (B) can be found at the associated Zenodo data repository (https://doi.org/10.5281/zenodo.5510697) in the file labeled 'S1_Raw_Images.pdf'.
