# Supplementary material for: Meiotic Cas9 expression mediates gene conversion in the male and female mouse germline
Source: PLoS Biol. 2021 Dec 23;19(12):e3001478. doi: 10.1371/journal.pbio.3001478 (PMC8699911; doi:10.1371/journal.pbio.3001478)
Supplement: S1 Table — (PDF) [file pbio.3001478.s008.pdf]

Primer name	Primer Sequence	Amplicon	Amplicon Length	Polymerase	Annealing Temp (˚F)	Elongation Time	Cycles	Seq Primer	notes
Chinchilla L3	GGGAGGAAAGGGTGCTTGAG	Chinchilla SNP	392 bp	MyTaq	60	1'	30	Chinchilla R1	
Chinchilla R1	CAGCAAGCTGTGGTAGTCGT								
Spo11-In-F	GAAGGGCATCGACTTCAAGGAGGAC		695 bp	MyTaq	60	1'	30		with Spo11-Out-R
Spo11-Out-F	TGCTGGTAACGCTGTGCTACCTTCT		502 bp						with Spo11-Out-R
Spo11-Out-R	ACCTCTCAAGGTTCGCTTGCTGTTT								
cc F1	TCAATGTCCCAGCTAGCAGAGGG	CopyCat	838 bp	MyTaq	60	1'	30		
Tyr HAR R2	GGTTCAAAAGCTTCCCAATCCT								
LHomF	CCGTTTGCCCATGCTGACTG	Lhom-F/R	1573 bp	MyTaq	59	1'30"	30	LHomF	
LHomR	TACTCGTCGGTGATCACGGC							LHomR	
1F	TGCTGGTAACGCTGTGCTACCTTCT	1-F/R	1042 bp	MyTaq	59	1'	30	1F	
1R	CCTCGGCCAGGTCGAAGTTG							1R	
2F	AAGAGCAGACGGCTGGAAAA	2-F/R	965 bp	MyTaq	59	1'	30	2F	
2R	AAGGCGGGCTTTCTCATTCC							2R	
3F	AAGAACCTGCCCAACGAGAA	3-F/R	923 bp	MyTaq	59	1'	30	3F	
3R	TGCAGCTGGGTGTTTTCCAC							3R	
4F	AACAGCCGCGAGAGAATGAA	4-F/R	915 bp	MyTaq	59	1'	30	4F	
4R	AAAATCCCGGCCCTTATCCC							4R	
5F	ACCGAGATTACCCTGGCCAA	5-F/R	911 bp	MyTaq	59	1'	30	5F	
5R	CTCTGGTGGATCAGGGTGGC							5R	
6F	ACCCTGACCAATCTGGGAGC	6-F/R	908 bp	MyTaq	59	1'	30	6F	
6R	TCTTTGCTCAGGGCGGACTG							6R	
7F	GAAGGGCATCGACTTCAAGGAGGAC	7-F/R	695 bp	MyTaq	59	1'	30	7F	
7R	ACCTCTCAAGGTTCGCTTGCTGTTT							7R	
RHomF	CCCGACAACCACTACCTGAG	Rhom-F/R	1700 bp	MyTaq	59	1'30"	30	RHomF	
RHomR	GGGGCCATCCTAACACCATA							RHomR	

S1 Table. Primer sequences and PCR conditions for each genotyping strategy.
