# Supplementary material for: Meiotic Cas9 expression mediates gene conversion in the male and female mouse germline
Source: PLoS Biol. 2021 Dec 23;19(12):e3001478. doi: 10.1371/journal.pbio.3001478 (PMC8699911; doi:10.1371/journal.pbio.3001478)
Supplement: S1 Raw Images — (PDF) [file pbio.3001478.s009.pdf]

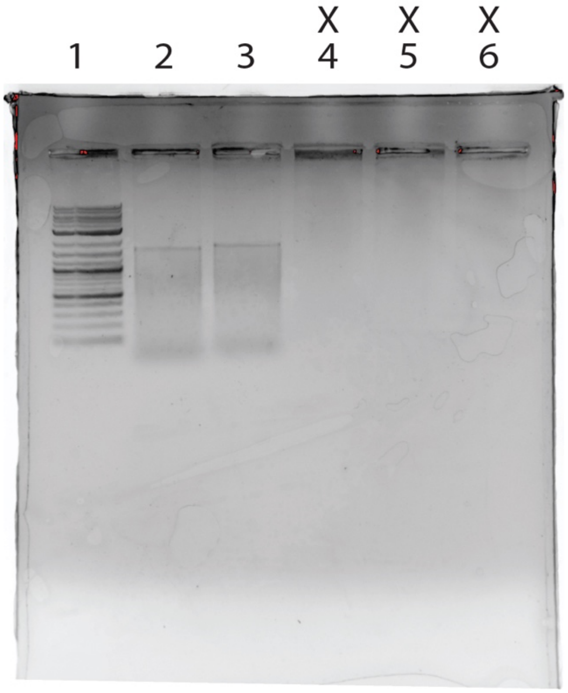

S1 Raw Images A.
Full uncropped gel from S1B Fig.

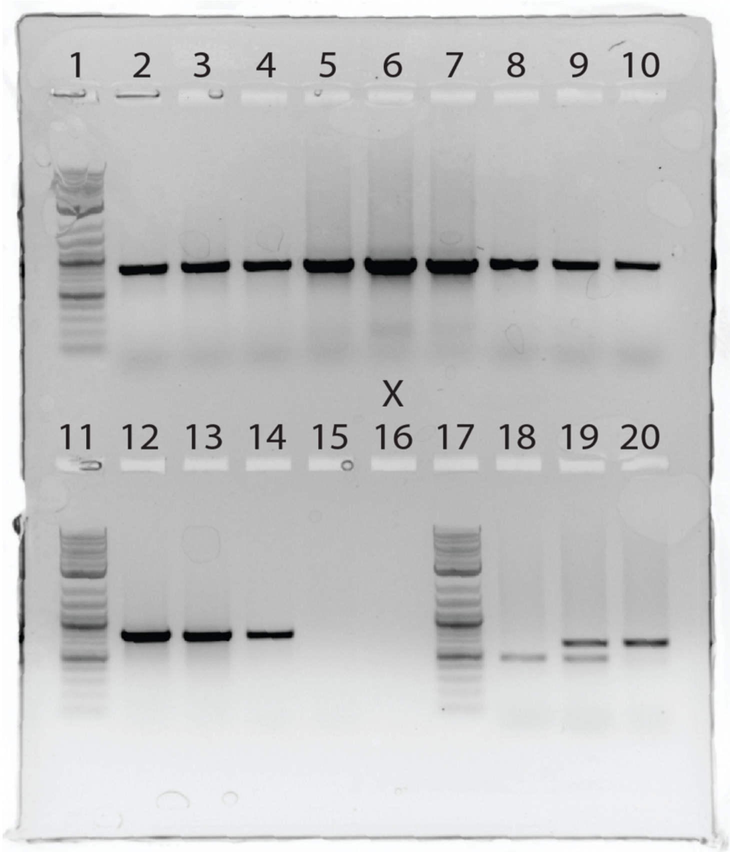

S1 Raw Images B.
Full uncropped gel from S6B Fig. (lanes 1-15) and from S7B Fig. (lanes 17-20)

Gel	Lane	Content
A	1	1kb Plus DNA Ladder (New England BioLabs, #N3200)
	2	S/+ mouse genomic DNA amplified with LHom-F/R
	3	S/+ mouse genomic DNA amplified with RHom-F/R
	4	Not loaded
	5	Not loaded
	6	Not loaded
B	1	1kb Plus DNA Ladder (New England BioLabs, #N3200)
	2	Male family 2a genomic DNA amplified with CC F1/Tyr HAR R2
	3	Male family 2b genomic DNA amplified with CC F1/Tyr HAR R2
	4	Male family 2c genomic DNA amplified with CC F1/Tyr HAR R2
	5	Male family 3 genomic DNA amplified with CC F1/Tyr HAR R2
	6	Male family 4 genomic DNA amplified with CC F1/Tyr HAR R2
	7	Male family 5a genomic DNA amplified with CC F1/Tyr HAR R2
	8	Male family 5b genomic DNA amplified with CC F1/Tyr HAR R2
	9	Male family 5c genomic DNA amplified with CC F1/Tyr HAR R2
	10	Female family 1 genomic DNA amplified with CC F1/Tyr HAR R2
	11	1kb Plus DNA Ladder (New England BioLabs, #N3200)
	12	Female family 4a genomic DNA amplified with CC F1/Tyr HAR R2
	13	Female family 4b genomic DNA amplified with CC F1/Tyr HAR R2
	14	Female family 4c genomic DNA amplified with CC F1/Tyr HAR R2
	15	WT genomic DNA amplified with CC F1/Tyr HAR R2
	16	Not loaded
	17	1kb Plus DNA Ladder (New England BioLabs, #N3200)
	18	WT genomic DNA amplified with Spo11-In-F/Spo11-Out-F/Spo11-Out-R
	19	S/+ genomic DNA amplified with Spo11-In-F/Spo11-Out-F/Spo11-Out-R
	20	S/S genomic DNA amplified with Spo11-In-F/Spo11-Out-F/Spo11-Out-R

Table of lane contents associated with images A and B.

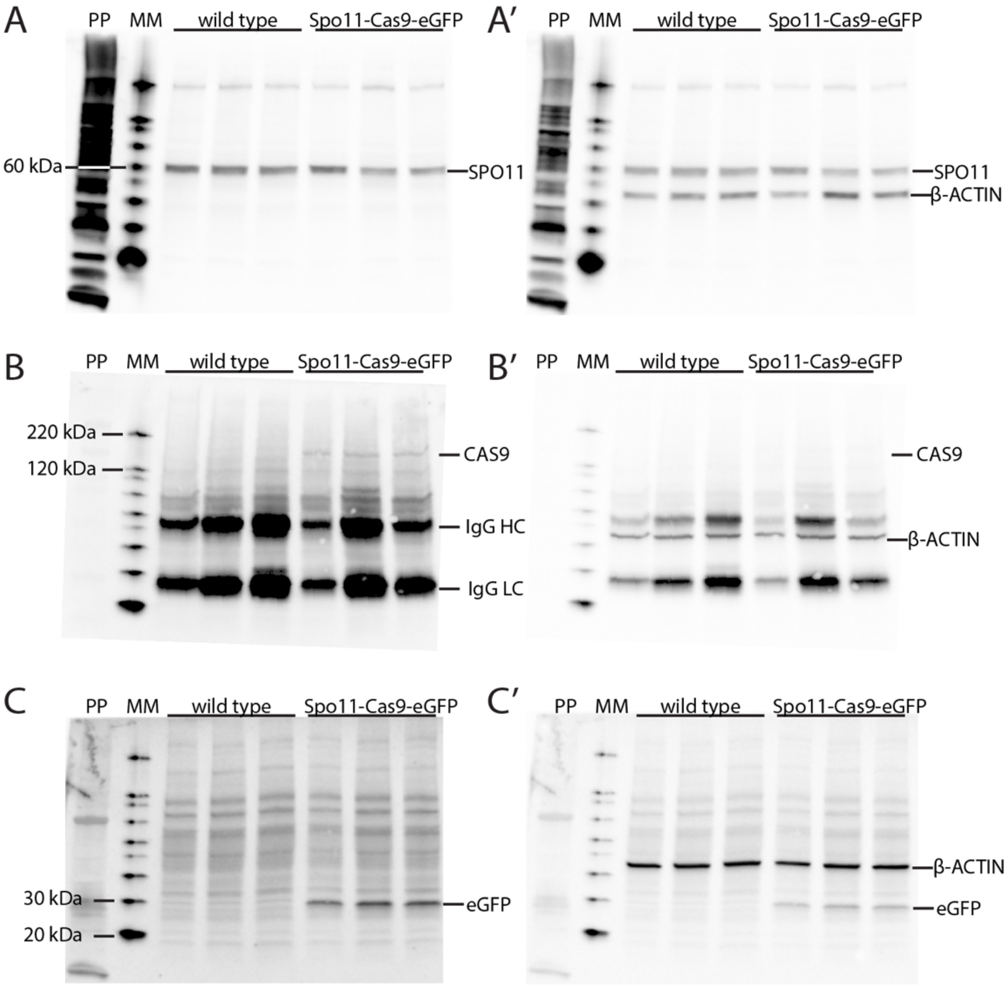

S1 Raw Image C.

Full uncropped gels that are the same as shown in S2 Fig.

(A-C) Western blot of (A) SPO11, (B) CAS9, and (C) eGFP on three wild type and three *Spo11*$^{Cas9\text{-}P2A\text{-}eGFP/+}$ adult testes. **(A'-C')** Serially-stained membranes adding anti β-ACTIN to detect the loading control. PP, Precision Plus Ladder; MM, MagicMark Ladder.
