# Supplementary material for: Meiotic Cas9 expression mediates gene conversion in the male and female mouse germline
Source: PLoS Biol. 2021 Dec 23;19(12):e3001478. doi: 10.1371/journal.pbio.3001478 (PMC8699911; doi:10.1371/journal.pbio.3001478)
Supplement: Attachment — Submitted filename: ReviewerResponse.pdf [file pbio.3001478.s010.pdf]

We thank all three Reviewers and the Academic Editor for their overall positive comments and for their valuable feedback. In our response below, we have placed each Academic Editor (AE) comment following the relevant Reviewer comment. We hope the Reviewers and Editor will agree that the additional data and revisions to the text have greatly improved this manuscript.

All three reviewers were concerned by infertility of the single Spo11-Cas9 homozygous male, which suggests SPO11 function was disrupted, despite our best efforts to preserve the locus. To address these concerns, we now show junction PCR with primers in the genome and in the synthetic knock-in allele that flank each homology arm and also tiled PCR with Sanger sequencing that spans the knock-in sequence. These data confirm that the allele was properly targeted. We also show Western blots of CAS9 and eGFP with each revealing a single band of appropriate size in Spo11-Cas9 heterozygous testes and not in wild type. We see no evidence of uncleaved polyprotein. We also did not observe a significant difference in the normalized level of SPO11 expression in Spo11-Cas9 heterozygous testes compared to wild type. In our response to Reviewer 1 below, we speculate on a possible explanation for reduced *Spo11* function that we chose not to include in the manuscript text, especially given this is a single infertile male. Regardless of unexpected presumed homozygous infertility, our observations using heterozygous animals support the conclusion that that 1) meiotic timing of Cas9 expression from the *Spo11* locus promotes male germline gene conversion 2) in a rather small proportion of all detectable repair types (~11%) and 3) in a proportion of all detectable repair types in females that is similar to our prior strategies. In our discussion, we suggest approaches to further refine meiotic timing, perhaps with the lesson to take extreme caution when designing knock-in alleles of essential endogenous loci.

Although not requested, we have replaced all incidences of ‘genotype conversion’ with ‘gene conversion’ in order to be consistent with our previous terminology in Grunwald et al. (2019) and with the revision of another manuscript concurrently in consideration elsewhere. Gene conversion is the term used in classical genetics to describe the “unidirectional transfer of genetic material from a 'donor' sequence to a highly homologous 'acceptor'” by transferring “genetic information from intact homologous sequences to regions containing double-strand breaks (DSBs)” [Chen *et al*. (2007) Gene conversion: mechanisms, evolution, and human disease. *Nature Reviews Genetics*].

Reviewer #1: In this manuscript, Weitzel and colleagues build upon their prior work in Cas9-mediated meiotic gene conversion to demonstrate a novel construct capable of inducing conversion in both the male and female germlines.

The experiments are logically planned and carried through, and the breeding data presented are sufficient to show that at least some of the final generation offspring carry the CopyCat and chinchilla alleles on the same chromosome (Fig 2D, right hand column). Although the numbers are low, this demonstrates conclusively that a DSB was formed on the target chromosome, which then underwent some kind of HDR event with the donor (CopyCat) chromosome.

My main technical query is in regard to characterising the Spo11-Cas9-GFP construct allele. Although they could not detect Cas9 by IF or WB, they could detect GFP by IF. Was GFP detectable by WB? This might give a clue as to the issues affecting protein expression. For example, if the P2A peptides are failing to cleave the expressed polyprotein, this could interfere with the folding and/or function of all three components and likely lead to substantial protein degradation. This would have the observed effects of a functional Spo11 knockout, low Cas9 expression, and GFP detectable only by IF and not by native fluorescence. A GFP WB would also provide better support for the claim that expression is higher in males - though I agree the supplementary figure is quite convincing in this regard.

We thank the Reviewer for their overall positive evaluation of our work and for requesting the GFP Western blot of Spo11-Cas9 samples. We now include Fig S2 that shows Western blot detection of GFP, as requested here, as well as SPO11 and CAS9 also discussed in greater detail below. Compared to wild type control testes, we detected a single unique band in each Spo11-Cas9 heterozygous testis that is the approximate size of GFP or CAS9, respectively, and no bands that would be consistent with remaining uncleaved polyprotein. We did not attempt the Western blots in meiotic ovaries because of the exceedingly small amount of embryonic ovarian tissue and difference in the relative proportion of germ cells and somatic cells in embryonic ovaries versus adult testes.

We hypothesize that a possible explanation for the Spo11-Cas9 homozygote infertility relates to the mechanism of P2A cleavage, which would have left 18 amino acids on the C-terminus of SPO11. In yeast, a single amino acid mutation near the C-terminus of Spo11 (Q376A) disrupts dimerization with Ski8. If this interaction is disrupted, then the entire Spo11 core complex dissociates [Bouuaert *et al*. (2021), Structural and functional characterization of the Spo11 core complex]. Although a 10xHis-3xFLAG tag on the C-terminus of Spo11 allows for core complex assembly [Bouuaert], and a single C-terminal FLAG tag has been shown to be tolerated for meiosis *in vivo* [Kugou *et al*. (2009), Rec8 guides canonical Spo11 distribution along yeast meiotic chromosomes], it is possible that the different sequence of 18 amino acids of P2A in our knock-in allele caused an unanticipated steric hindrance between Spo11 and Ski8, which could disrupt the core complex necessary for meiosis. Our interpretation is highly speculative, however, and given the tolerance for other additional C-terminal amino acids, we chose not to include this in the manuscript text.

Although not necessary for this paper, an obvious double check for all these results would be to use the a Spo11-Cre allele in conjunction with LSL-Cas9 to see if this gives higher expression of Cas9 in the desired cell types.

AE: This might serve as good controls but I don't think it is absolutely necessary.

We agree this will be a very interesting experiment to pursue in the future, and we thank the reviewer for explicitly stating that this is not necessary for the current

paper. The Spo11-Cre line deposited with the Jackson Laboratory is cryopreserved and would require 3+ months for recovery to obtain animals to begin our crossing strategy.

The remainder of the discussion is generally fine, but neglects the possibility that conserved alleles may have nevertheless been cut, but repaired via HDR from the sister chromatid. Although this is a rare possibility, it does occur for at least some breaks, perhaps particularly those occurring in late pachytene, diplotene or in secondary spermatocytes. Given the stronger expression of the male meiotic Cas9 in late pachytene/diplotene, this should be mentioned as a possibility.

We thank the reviewer for pointing out this possibility. We have revised the third paragraph of the Discussion (Page 9, lines 11-15) where we argue that we think we can assume most CRISPR-induced DSBs are repaired ‘non-conservatively’ for the sake of our models. We have added this definitive possibility that some small fraction of DSBs are conservatively repaired by HR from the sister chromatid.

Reviewer #2: Weitzel et al present a well-written manuscript claiming:

1. meiotic expression of Cas9 mediates genotype conversion in the male as well as in the female germline.
2. Cas9 expression levels may govern genotype conversion frequencies.
3. expression of Cas9 during meiosis I allows genotype conversion in the male and female germlines.

I find the ideas in the paper to be exciting and the potential applications (pest control gene drives, simplification of breeding strategies) to be high impact. However, I feel that the paper does not meet the scope of a 'Discovery Report' provided to me during the review process. The authors' argument is that they have a preliminary approach for germline genotype conversion and that this could be improved by increasing Cas9 expression or by refining timing of Cas9 expression. In my opinion, the authors do not do enough to justify future research in these directions and do not rule out alternative explanations. My recommendation is thus to revise the manuscript. My specific comments are below.

AE: I do not fully agree with this point. May we can ask for a better support with substantial addition of data? But for the purpose of ‘Discovery Report’, the current manuscript might suffice. Nevertheless, I still prefer to go for more conventional format if at all possible.

Upon evaluating the reviewer reports and the manuscript once again, the editorial team determined the manuscript would be better suited as a ‘Methods and Resources’ article. We have therefore submitted our revision as such. The manuscript is still organized in a conventional format.

Major Concerns

1. I find the phenotype of the homozygous mouse to be concerning. To me, this implies that Spo11 in the fusion construct is damaged or repressed due to the extended transcript. The genotyping in Figure S1 does not rule out that this could have occurred. It would be nice to see better validation that the original construct inserted successfully into the genome (junction PCR and sequence validation, for example). It would also be nice to see some validation that Spo11 expression levels are relatively normal in the context of the -P2A-Cas9-P2A-eGFP fusion. This seems challenging in the context of tissue, but might be addressable by IF. My issue here is that the authors do not rule out the possibility that something intrinsic to their construct is the cause of lower than expected genotype conversion efficiency.

We thank the Reviewer for requesting additional validation of the Spo11-Cas9 allele. In this revision, we show that the left and right homology arms can each be amplified using one primer embedded in the genome outside of the homology arm and a primer within the synthetic knock-in allele. We have also Sanger sequenced overlapping amplicons that span the full-length locus, and we deposited the confirmed sequence of this knock-in allele to Genbank.

We have also now performed Western blot of SPO11 protein expression in wild-type and Spo11-Cas9 heterozygous testes. Normalized to the level of beta-ACTIN loading control, we detect no significant difference in the level of SPO11 expression. CAS9 and GFP were also individually detectable with no evidence of failed polyprotein cleavage. We discuss a speculative alternative explanation for presumed homozygous infertility in response to Reviewer 1 above.

2. The claim about Cas9 expression levels is interesting but needs to better supported to justify further investigation. One alternative explanation is some aspect of the original construct reduces Cas9 expression, as discussed above. Another alternative explanation would be silencing of the gRNA in the CopyCat cassette. I would want to see data indicating these alternative explanations were unlikely. Data (obtained using similar Cas9 constructs) suggesting that Cas9 levels higher than those measured here increased genotype conversion would also be conclusive.

We appreciate this comment and the Reviewer's concern. Our interpretation is that the level of Cas9 level expression is primarily important for DSB frequency and therefore total detectable repair (indel + HDR) rather than the proportion of DSB repair by gene conversion. This is based on our observation in this and in our prior paper that a presumed higher level of expression from the constitutive CMV promoter resulted in 100% DSB repair (indels) in males while transient and presumably lower expression from the *Spo11* locus results in 29% DSB repair (indel + HDR). This is also consistent with literature that established the mass action kinetics of Cas9.

In our previous work, all animals that expressed constitutive Rosa26-Cas9 and *Tyrosinase*^{CopyCat} and all offspring of males that expressed germline-restricted Vasa-

Cre;LSL-Cas9;Tyr^{CopyCat} were white (indel or HDR) indicating there is sufficient gRNA expression for highly efficient DSB formation. In the experiments detailed here, we used the same source of gRNA. The new variable is the source of Cas9 expression, which we think is cumulatively lower due to transient low expression of native Spo11 (versus the constitutive CMV promoter) or to something intrinsic to our knock-in allele. Although we cannot rule out the possibility that gRNA expression may have changed in the stable Tyrosinase^{CopyCat} line, all mice continue to express mCherry from the same locus.

We now have an antibody that detects Cas9 in Western blots (thank you!), but we unfortunately no longer maintain the Vasa-Cre or Rosa26-LSL-Cas9 lines used for germ line strategies in Grunwald et al (2019), and both lines are now cryopreserved at the Jackson Laboratories. The constitutive Rosa26-Cas9 line is available as live mice, but we are concerned that expression in all germ *and* somatic cells will confound interpretation of the expected higher expression in this line compared to Spo11-Cas9.

3. The claim that Meiosis I expression of Cas9 is important is compelling, but I find the definition of this timing in the manuscript to be somewhat circular. The authors cite expression patterns of Spo11 during meiosis, then use the expression of their GFP construct (driven by the Spo11 promoter) to define meiotic populations. At a minimum, the authors should use independent markers and demonstrate that their GFP expression patterns match expectations. Ideas in the discussion section to use promoters that "drive strong expression with less temporal specificity and to chemically control the timing of Cas9 activity" would also address this concern.

AE: The authors have made a knockin allele, so I am not concerned as much as this reviewer. I'd rather agree with Rev1 that the authors may use WB or IP/WB to detect the expression of Cas9 and eGFP in the knockin animals.

We thank the Reviewer for pointing out that we may not have been clear in our previous description of native SPO11 expression. We now provide more detail from the cited references showing how these meiotic cell populations were defined *prior* to SPO11 detection (Page 4, lines 8-10). We also added detail previously only in the figure legend that describes how we define spermatogonia and spermatocytes in IF by chromatin compaction state (DAPI) and by VASA protein expression, which is relatively lower in the outer ring of late-stage spermatagonia than within spermatocytes (inset Figure1g). If all VASA+ cells were eGFP+ in all tubules, we would have to conclude that the knock-in allele was erroneously initiated in spermatogonia. We think the identification of outer perimeter VASA+ cells that are not eGFP+ supports our conclusion that Spo11$^{Cas9\text{-}P2A\text{-}eGFP}$ is not expressed in late stage spermatogonia as they initiate VASA expression.

Comments

1. Cas9 antibodies are notoriously awful. Most people in the field use epitope tagged Cas9 for this reason. Some labs have had luck with Cell signaling 14697 and this clone from other vendors.

AE: This is a helpful comment. I'd definitely try to use a few antibodies to test the knockin allele with by WB or some other method than IF, which suffers a lot from autofluorescence from the mouse tissues.

We are very grateful to the Reviewer for this suggestion, which also helped us to address the first point raised by Reviewer 1 above. We obtained the recommended Cell Signaling antibody and implemented a protocol that allowed us to detect Cas9 expression using this mouse primary antibody in mouse tissues. Although mouse immunoglobulin bands are prominent even after blocking with mouse Fab fragments, we are indeed able to detect Cas9 expression in Spo11-Cas9 heterozygous testes. This antibody reveals a single unique band of size corresponding to Cas9 in Spo11-Cas9 testes that is not present in wild-type testes confirming that the P2A cleavage occurred correctly in a majority of translation events.

Reviewer #3: This manuscript describes additional "gene conversion" experiments from the group that previously published the first evidence for "gene drive" activity in mice (Grunwald et al). This research is highly topical - not least because of the potential for genetic biocontrol applications as well as generation of homozygous multi-allele disease models with greater efficiency. The study is very focussed - essentially comprising a single experiment that investigates germline HDR/EJ efficiency from a new Cas9-driver (Spo11-KI) allele. The elegant mating scheme used to detect gene conversion (which has the distinct advantage of a marked recipient chromosome) has been used previously (Grunwald et al).

The manuscript is well written and the figures clear and informative. The following suggestions (many of which are minor) should be considered to improve the manuscript.

We are grateful to the Reviewer for their positive and constructive comments.

1. P2 Line 34. The statement of "72%" doesn't give a sense of the inter-individual variability seen previously in the Grunwald et al paper - suggested clarifying.

Thank you for pointing this out. We have clarified this sentence to now read, "The frequency of allele transmission increased from 50%, predicted by Mendelian inheritance, to <u>an average of</u> 72% <u>among offspring of five females</u> after CRISPR/Cas9-mediated genotype conversion in the germline."

2. P3 line 4 "achieve timing" - could be expressed more clearly.

We have replaced this phrasing with "to control the timing of Cas9 expression"

3. P3 Line 18 "SPO11" (ie. in CAPS) as the protein is being referred to.

Thank you. We have corrected the nomenclature so that the protein is reference in all caps.

4. P3 line 27 Although this issue is addressed in the discussion a caveat here is that DSB repair may be perfectly repaired and therefore not detected. Perhaps use "detectable DSB repair".

We agree, and we and have revised the phrasing as requested.

5. P5 line 31 - P7 line 4; The description of the mating scheme is slightly confusing because the Spo11-Cas9/+; Tyr ch/ch mice described in p7 line 2 cannot be generated by crossing Spo11-Cas9/+ with Tyr ch/ch mice (i.e. only Tyr ch/+ can be generated). Also, suggest including the male origin of the Spo11-Cas9 allele on Fig. 2.

We thank the reviewer for alerting us to the fact that we unintentionally omitted the step where we backcrossed some *Spo11-Cas9/+;Ch/+* mice to Tyr^{ch} in order to homozygose the marked recipient chromosome. These are the mice represented in Figure 2. We have added a sentence to the text to make this clear (Page 6, lines 14-15). We have also added ‘male’ and ‘female’ symbols to Figure 2 to illustrate the direction of the cross.

6. Despite the efforts not to reduce SPO11 activity, it appears that the P2A linker KI approach may have ablated/reduced SPO11 function. However, the only data provided to assess this is from a single HOM male animal (Fig S2). It would obviously be informative to include more data from HOM breeders if available. Also, corroborating evidence of reduced Spo11/SPO11 expression in HOM and HET Spo11-Cas9 mice would be very useful to include in the manuscript.

AE: I’d also welcome any additional data but this is again optional. I wish that the authors discuss this point well in the discussion.

This is a very valuable suggestion. Although we established matings of our limited number of Spo11-Cas9 heterozygous male and female mice as requested in June, we obtained no more homozygous individuals. We did obtain more heterozygous males allowing us to euthanize some of the males we have at older breeding ages (~6 months). Testis specimens of these animals are now in the Western blots of Fig S2 demonstrating protein expression in wild type and Spo11-Cas9 heterozygous animals. We did not detect a significant difference in SPO11 expression when normalized to beta-ACTIN expression. We also detect no evidence of polyproteins that would indicate incomplete peptide cleavage. We had no protein lysate from a homozygous testis sample for these blots.

7. Given that SPO11 has a role in homologous recombination repair, if indeed SPO11 function has been reduced, it is possible that the Spo11-Cas9 HET mice

used in the gene conversion test cross may have a reduced capacity for homologous recombination (aka HDR) - which is the mechanism used for gene conversion. This possibility should be acknowledged.

AE: Good point!

We thank the reviewer for pointing out this possibility, which we now acknowledge on Page 11, lines 26-29, together with a reminder than Spo11 is haplosufficient.

8. Did you look for any evidence of "Cas9 carry-over" into the gametes (i.e. indel mutations in the Tyr-null allele)? Would these be indistinguishable from the "EJ" group? (Fig. 2D)

This is an excellent question that we have considered in the past. Indels caused by carryover of Cas9 would be indistinguishable from the EJ group if mutagenesis was early and efficient in the early zygote (entirely white mice). However, given the low frequency of detectable repair in offspring of males and of females in the Spo11-Cas9 strategy, we would expect to see some degree of coat color mosaicism if EJ did not occur in the germline and instead occurred by Cas9 carryover in the zygote. All offspring of these crosses were either grey (functional Tyr^{ch} allele over Tyr^{null}) or white (disrupted Tyr^{ch} allele over Tyr^{null}). This seems consistent with the brief duration of Spo11 expression during oogenesis and spermatogenesis that is not detected in mature sperm or eggs.

9. The discussion is quite speculative. The model is based on estimation of expression levels and timing from different studies and techniques rather than direct comparison. That being said, I think it is a reasonable and likely correct interpretation of the available data and represents a valuable addition to our collective thoughts about of how gene conversion occurs and how it may be improved. As far as I am aware, careful comparison of Cas9 expression has not been performed even in Diptera (where gene conversion rates are generally much higher).

We agree with the Reviewer that our model is 'quite speculative', and we are grateful the Reviewer agrees that the model is 'reasonable' given the available data and 'valuable' to shape future research.

10. Fig. S4 is only referred to in the Methods section. Could the authors please indicate if there was a perfect correlation between Cherry expression and a Tyr-Copycat allele-positive genotype.

Fig S4 (now Fig S5) is referenced in the main text on Page 6, line 27 as an example of observed mCherry fluorescence. Yes, there is a perfect correlation between mCherry expression and $Tyr^{Copycat}$ genotype in this work and in Grunwald et al, 2019.
